# An Examination of the Ethnobotanical, Phytochemical, Antimicrobial, and Biological Properties of *Zygophyllum coccineum*, Emphasizing Its Potential as a Valuable Forage Shrub

**DOI:** 10.3390/life15040661

**Published:** 2025-04-17

**Authors:** Fawziah M. Albarakaty, Mashail N. AlZain, Rehab M. A. El-Desoukey

**Affiliations:** 1 Department of Biology, Faculty of Science, Umm Al-Qura University, P.O. Box 715, Makkah 21955, Saudi Arabia; fmbarakati@uqu.edu.sa; 2 Department of Biology, College of Science, Princess Nourah Bint Abdulrahman University, P.O. Box 84428, Riyadh 11761, Saudi Arabia; mnalzain@pnu.edu.sa; 3 Microbiology and Immunology Department, National Research Centre, Giza 12622, Egypt; 4 Natural and Applied Sciences Department, Scientific Departments in Afif, Shaqraa University, Shaqraa 11961, Saudi Arabia

**Keywords:** antimicrobial activity, *Zygophyllum coccineum*, antioxidant, natural extracts, cytotoxicity, ethnobotanical, antibiotic resistance, veterinary, phytochemical

## Abstract

The growing issue of antimicrobial resistance poses a significant challenge for microbiological research, driving the need for alternative antibiotics with minimal side effects. *Zygophyllum coccineum*, commonly referred to as “Tebtab” in Arabic and “Red Spinepod” in English, has traditionally been utilized as forage for camels and ruminants. While its antimicrobial activity against human pathogens has been documented, its efficacy against animal pathogens remains underexplored. This study aims to evaluate the phytochemical composition, biological activities, and antimicrobial potential of water and organic-solvent extracts of *Zygophyllum coccineum* against a range of reference microbial strains and animal pathogens. The findings revealed that all extracts exhibited notable antimicrobial, antioxidant, and cytotoxic activities attributed to their bioactive constituents. Among them, the ethyl acetate extract displayed the strongest antimicrobial effects against bacterial and fungal strains. Additionally, this extract demonstrated the highest antioxidant capacity and showed promising cytotoxic activity against lung (A549) and breast (MCF-7) cancer cell lines. These results underscore the potential of *Zygophyllum coccineum* as a valuable natural resource for developing antimicrobial, antioxidant, and cytotoxic therapies for applications in both human and veterinary medicine.

## 1. Introduction

Microbial infections remain a leading cause of global morbidity and mortality, driven by the alarming rise in antimicrobial resistance (AMR) among pathogens. This resistance is primarily attributed to the widespread misuse and overuse of antimicrobial agents, rendering many conventional treatments ineffective and posing a significant challenge to global public health. In veterinary medicine, AMR is a critical concern, as animal-derived pathogens contribute to zoonotic diseases and the spread of resistant strains to humans and within the environment [1,2]. This scenario underscores the urgent need for alternative antimicrobial agents that are both effective and sustainable.

Natural products derived from plants have historically served as a foundation for modern medicine, with over half of today’s pharmaceuticals originating from natural sources [3]. Plants are a rich reservoir of bioactive compounds with diverse pharmacological properties, including antimicrobial, antioxidant, and anticancer activities. In arid regions, particularly in Saudi Arabia, native plants that have adapted to harsh environmental conditions have been traditionally utilized for their medicinal properties. Despite their potential, many of these plants remain underexplored as to their therapeutic applications [4,5].

**Figure 1:**
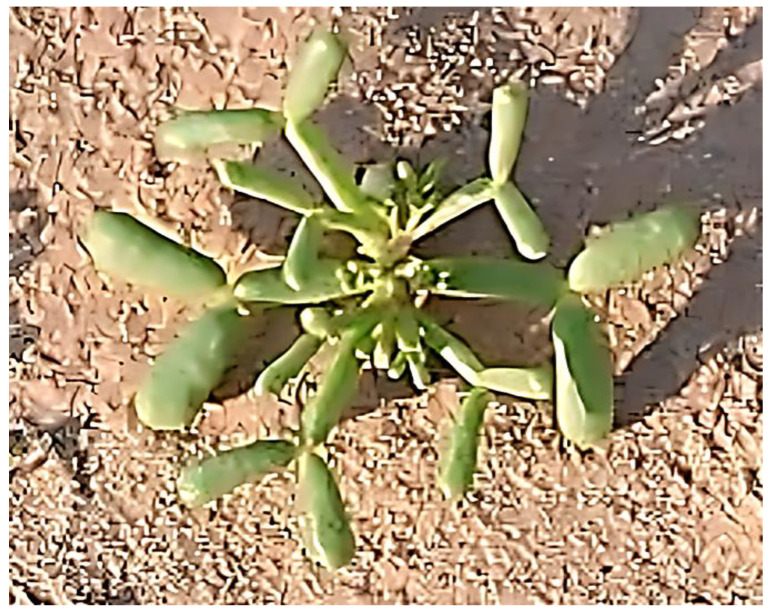
*Zygophyllum coccineum* (Tebtab).

*Zygophyllum coccineum* L., commonly known as Tebtab, is a subshrub, belonging to the *Zygophyllaceae* family, with remarkable ecological adaptability (Figure 1). It thrives in diverse soil compositions and saline habitats, displaying a strong tolerance for heavy-metal stress. This plant has a wide range of traditional medicinal applications, including treatments for skin, stomach, and liver ailments, as well as conditions like rheumatism, gout, hypertension, and flatulent colic. It is also recognized for its anthelmintic, anti-inflammatory, and antidiabetic effects, which are attributed to its rich composition of flavonoids and phenolic acids [6,7,8]. Additionally, *Z. coccineum* plays an essential role in traditional veterinary medicine, where it is used for livestock and camels and as a therapeutic agent for conditions like arthritis [6,9].

Research highlights the plant’s notable antimicrobial, cytotoxic, antihypertensive, and anti-inflammatory properties. Its ethyl acetate extract demonstrates significant efficacy against bacterial and fungal strains, as well as cancer cell lines, corroborating its traditional uses. Genetic studies reveal substantial diversity in *Z. coccineum*, which enables it to flourish across Egypt and Saudi Arabia in saline and sandy habitats [8,9,10,11].

While *Z. coccineum* has been traditionally used in Arabic medicine, its antimicrobial properties, particularly against animal-derived pathogens, remain insufficiently explored. Investigating its therapeutic potential addresses the AMR challenge in veterinary pathogens and expands the repertoire of sustainable natural alternatives used for combating zoonotic diseases.

This study aims to comprehensively evaluate the phytochemical, antimicrobial, antioxidant, and cytotoxic properties of *Z. coccineum*, with a novel focus on its efficacy against animal-derived pathogens. Unlike previous studies that predominantly targeted human pathogens, this research explores its veterinary applications, addressing a critical gap in the literature. By providing detailed insights into the plant’s bioactive compounds and their pharmacological effects, this work highlights the broader implications of *Z. coccineum* as a valuable yet underutilized resource in natural medicine.

## 2. Materials and Methods

### 2.1. The Study Employed the Following Materials and Reagents:

The solvents and standards (analytical grade) employed in this study were obtained from Sigma-Aldrich. The investigation employed a BȔCHI Rota-vapor R205 rotary evaporator, manufactured in Switzerland, alongside an HPTLC system provided by Eike Reich/CAMAG-Laboratory, also based in Switzerland. TLC aluminum plates, measuring 10 × 5 cm^2^ and possessing a thickness of 0.2 mm, were pre-coated with silica gel 60 F 254 and provided by E. Merck Ltd., Mumbai, India. For GC-MS analysis, an Agilent Technologies 7890B GC system from Santa Clara, CA, USA, was used, featuring an auto sampler that injected 0.7 µL of the sample. Product identification was conducted using the integrated software from NIST MS. The cell lines used in our research were obtained from the DSMZ Leibniz Institute (German Collection of Microorganisms and Cell Cultures) in Braunschweig, Germany. Specifically, the cell lines included the A549 lung cancer and MCF-7 breast cancer lines.

### 2.2. Bacterial and Fungal Strains

The study utilized several bacterial and fungal strains, including animal-derived pathogenic isolates such as *Klebsiella pneumoniae*, *Enterococcus faecium*, *Pseudomonas aeruginosa*, *Staphylococcus aureus*, *Escherichia coli*, *Salmonella typhimurium*, *Bacillus cereus*, *Streptococcus pyogenes*, and *Candida albicans*, obtained from apparently healthy and diseased animals in large animal and poultry farms in Giza, Egypt. Additionally, reference microbial strains were sourced from Sigma-Aldrich, including *Enterococcus faecalis* ATCC 29212, *Salmonella typhimurium* ATCC 14028, *Escherichia coli* ATCC 25922, *Staphylococcus aureus* ATCC 29213, *Candida tropicalis* ATCC 66029, and *Candida albicans* ATCC 60193.

### 2.3. Plant Material Collection and Preparation

#### 2.3.1. Collection of Shrub

The aerial parts of *Zygophyllum coccineum* were collected during the flowering season, in April 2022, from Quwai’ Vale in Al Quwai’iyah, a prominent province near Riyadh, Saudi Arabia. This region is characterized by its expansive flat terrain, bordered by mountains on three sides. The western area is known for its active supply chain, while the eastern section, called Hadba, along with the Jala desert, represents the pinnacle of the Arabian Shield geological formation. Al Quwai’iyah holds historical importance as a crossroads between ancient pyramids and calcareous limestone formations. The Al Quwai’ Valley extends west to east into the Hadba desert and is rich in wild herbs and shrubs, commonly used for grazing, fuel, and traditional medicine. Ethnobotanical studies have documented numerous plant species in the area, many of which remain underutilized despite their significant potential for local and global applications, demonstrating resilience to harsh environmental conditions and contributing to nutrition and natural healing practices.

#### 2.3.2. Ethnobotanical Verification

The classification and ethnobotanical documentation of the *Z. coccineum* were conducted by Dr. Mashail Nasser AlZain, Assistant Professor of Plant Ecology at the Biology Department, Princess Nourah bint Abdulrahman University, Riyadh. This was corroborated by studies referenced in [4] and supported by data deposited in the herbarium of the Faculty of Pharmacy, King Saud University, Riyadh, under voucher number 15803.

### 2.4. Extraction Processes

#### 2.4.1. Water Extraction

Aqueous extracts of the aerial parts of *Zygophyllum coccineum* were prepared using both cold and hot extraction techniques. For the cold extraction, the plant material was infused in water at room temperature, while for the hot extraction, the material was subjected to decoction in water heated to 100 °C. The infusion and decoction processes were carried out according to the protocol described in [12]. After extraction, the resulting solutions were filtered to remove plant debris and stored under appropriate conditions until further analysis.

#### 2.4.2. Organic-Solvent Extraction

The desiccated aerial parts of *Z. coccineum* were subjected to extraction using various organic solvents, including methanol, ethyl acetate, chloroform, n-hexane, acetone, n-butanol, and ether. The extraction procedure was performed as per the methodology detailed in [13]. Each solvent was chosen to target specific classes of phytochemicals based on their polarity. The plant material was soaked in the respective solvent, followed by agitation and filtration. The filtrates were then concentrated under reduced pressure using a rotary evaporator and stored for subsequent bioactivity and chemical composition analyses.

### 2.5. Phytochemical Analysis

#### 2.5.1. Evaluation of Phenolic Compounds

The total phenolic contents of the crude methanol extracts were quantified using the Folin–Ciocalteu colorimetric assay, following the methodology outlined in [13]. Briefly, an aliquot of the methanol extract was mixed with the Folin–Ciocalteu reagent, followed by the addition of sodium carbonate solution to neutralize the reaction. The mixture was incubated at room temperature, and the absorbance was measured spectrophotometrically at a specific wavelength. Gallic acid was used as a standard for calibration, and the phenolic content was expressed as milligrams of Gallic acid equivalents (mg GAE) per gram of extract.

#### 2.5.2. Determination of Total Flavonoid Content

The total flavonoid content in the crude methanol extracts was assessed using a colorimetric method described in [14]. The extract was mixed with an aluminum chloride solution, which formed a complex with flavonoids, resulting in a color change. The mixture was incubated at room temperature, and the absorbance was recorded at a specific wavelength using a spectrophotometer. Quercetin was employed as the reference standard, and the flavonoid content was expressed as milligrams of quercetin equivalents (mg QE) per gram of extract.

#### 2.5.3. GC-MS Analysis

Component identification was conducted using Gas Chromatography–Mass Spectrometry (GC-MS) with an Agilent Technologies DB-5 MS capillary column (30 m × 0.25 mm internal diameter × 0.25 µm phase thickness). Helium served as the carrier gas, at a flow rate of 1 mL/min. The inlet temperature was set to 250 °C in split mode (50:1). The oven temperature was programmed to increase from 50 to 250 °C over 50 min. Mass spectrometry settings included a mass range of 40–500 g/mol, a scan speed of 1.56, a solvent delay of 9 min, and a source temperature of 230 °C, as per the methodology in [15].

### 2.6. Biological Assays (Antioxidant, Antimicrobial, and Anticancer Evaluations)

#### 2.6.1. Antioxidant Activity Evaluation

The antioxidant capacity of the *Z. coccineum* extracts and their fractions was evaluated using an ABTS radical cation scavenging assay based, with some modifications, on the protocol described in [16]. ABTS radical cations were generated by reacting the ABTS solution with a suitable oxidizing agent. The extracts were then mixed with the prepared ABTS radical solution, and the decrease in absorbance was measured at a specific wavelength using a spectrophotometer. The antioxidant activity was expressed as IC50, indicating the concentration of the extract required to scavenge 50% of the ABTS radicals.

#### 2.6.2. Cell Viability Assay

The cytotoxicity and cell viability effects of *Z. coccineum* extracts were assessed using the MTT assay, which was based on the protocol outlined in [16]. In this assay, cells (A549 (lung cancer) and MCF-7 (breast cancer)) were seeded into 96-well plates and treated with different concentrations of the extracts. After the incubation period, MTT reagent (a yellow tetrazole) was added to each well. Viable cells metabolized the MTT into insoluble formazan crystals, which were dissolved using a solubilization solution. The absorbance of the resultant solution was measured at 570 nm using a microplate reader. The cell viability was expressed as a percentage relative to untreated control cells.

#### 2.6.3. Antimicrobial Activity Evaluation

The antimicrobial activity of *Zygophyllum coccineum* extracts was assessed using the agar well diffusion technique. In this method, Mueller Hinton Agar (MHA) plates were prepared and inoculated with bacterial and fungal suspensions, standardized to the appropriate concentration (e.g., the 0.5 McFarland standard). Wells of uniform size were created in the agar using a sterile borer, and each well was filled with a specific volume of the extract at a predetermined concentration. The plates were incubated at the appropriate temperature (e.g., 37 °C for bacterial strains and 25–28 °C for fungal strains) for 24–48 h. After incubation, the diameters of the zones of inhibition around the wells were measured in millimeters to determine the antimicrobial efficacy of the extracts, following the protocol described in [16].

#### 2.6.4. Determination of Minimum Inhibitory Concentration (MIC)

The minimum inhibitory concentration (MIC) of the *Z. coccineum* extracts was determined using a broth microdilution method, as described in [16]. Serial dilutions of the extracts were prepared in a 96-well microplate to obtain a range of concentrations. Each well was inoculated with a standardized microbial suspension. Positive controls (microorganisms without extract) and negative controls (media without microorganisms) were included to ensure the validity of the results. The plates were incubated at optimal growth conditions for the respective microorganisms (e.g., 37 °C for bacteria and 25–28 °C for fungi) for 24–48 h. The MIC was defined as the lowest concentration of the extract that visibly inhibited microbial growth, as indicated by the absence of turbidity in the wells.

#### 2.6.5. Determination of Minimum Bactericidal/Fungicidal Concentration (MBC/MFC)

The minimum bactericidal concentration (MBC) or minimum fungicidal concentration (MFC) was determined by subculturing aliquots from the MIC wells that showed no visible growth onto agar plates suitable for the specific microorganism (e.g., Mueller Hinton Agar for bacteria and Sabouraud Dextrose Agar for fungi). The plates were incubated under optimal conditions for 24–48 h. The MBC/MFC was identified as the lowest concentration of the extract that resulted in no visible colony formation, indicating complete microbial eradication. These procedures were conducted by the methodologies described in [16].

### 2.7. Statistical Analysis

All tests were conducted in triplicate, and the results were analyzed using one-way analysis of variance (ANOVA) with Origin Pro 8.5 software to calculate means and standard deviations. The least significant difference (LSD) test was employed to compare means, with a significance level of *p* ≤ 0.05. Correlation coefficients and inter-variable relationships were explored using SAS software version 9.4 (SAS Institute Inc., Cary, NC, USA). IC50 values were determined using Microsoft Excel (with the Solver Add-in).

## 3. Results

The present study focused on evaluating the phytochemical composition and antioxidant, cytotoxic, and antimicrobial properties of *Zygophyllum coccineum* extracts, a resilient desert shrub with significant traditional and therapeutic applications. This plant, known for its ability to thrive in arid and saline environments, is a source of bioactive compounds, including phenolics, flavonoids, and fatty acids, which are pivotal to its pharmacological potential. *Z. coccineum* has been traditionally used as a grazing herb and for its medicinal properties, including antifungal, anti-inflammatory, and antihyperglycemic effects. The results presented here provide new insights into its phytochemical richness and biological activities.

### 3.1. Determination of Phenolic and Flavonoid Content of Zygophyllum coccineum

The data in Table 1 indicates that the total phenolic content of the crude extract from *Zygophyllum coccineum* is 156.15 mg GAE/g of dry extract, while the flavonoid concentration is measured at 18.5 mg QE/g of dry extract.

### 3.2. GC-MS Profile of Zygophyllum coccineum

The Gas Chromatography–Mass Spectrometry (GC-MS) analysis of the methanol extract from *Zygophyllum coccineum* identified 20 putative phytochemical compounds. These compounds were characterized based on their retention times (RT), molecular weights (MW), peak areas (indicating their relative concentrations), and quality of identification.

Key findings from Table 2 include the following:

- Undecane (RT = 5.927 min): A volatile, low-molecular-weight hydrocarbon detected with moderate abundance (Area = 249,400 Ab*s). Its early elution suggests its volatile nature.
- n-Hexadecanoic acid (RT = 12.338 min): A commonly occurring fatty acid in plant extracts with known biological properties. It was relatively abundant (Area = 979,229 Ab*s), and was identified with a high confidence score of 99.
- Cyclopentane-3′-spirotricyclo [3.1.0.0(2,4)] hexane-6′-spirocyclopentane (RT = 13.552 min): The most abundant compound in the extract, accounting for the majority of the phytochemical content (Area = 22,662,616 Ab*s).
- Bergamotol, Z-α-trans- (RT = 14.828 min): Another significant compound, detected with moderate abundance (Area = 1,715,991 Ab*s).

**Figure 2:**
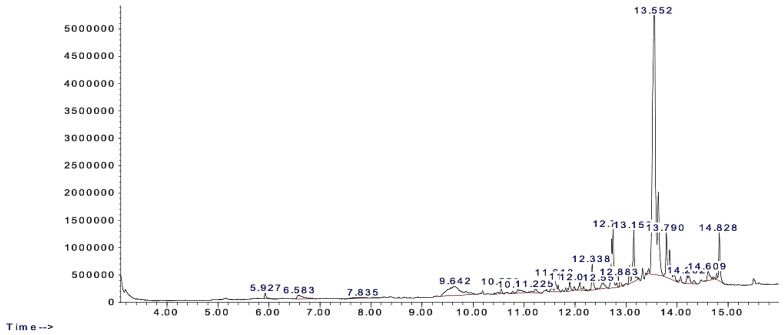
Chromatogram of crude methanol extract of *Zygophyllum coccineum*.

The GC-MS chromatogram (Figure 2) provides a detailed visualization of the elution profiles for these compounds. Each peak represents an individual compound, with the area under the curve reflecting its relative concentration. Major peaks correspond to Undecane (RT = 5.927 min), n-Hexadecanoic acid (RT = 12.338 min), and the dominant compound Cyclopentane-3′-spirotricyclo [3.1.0.0(2,4)]hexane-6′-spirocyclopentane (RT = 13.552 min).

### 3.3. Antioxidant Scavenging Activity

**Figure 3:**
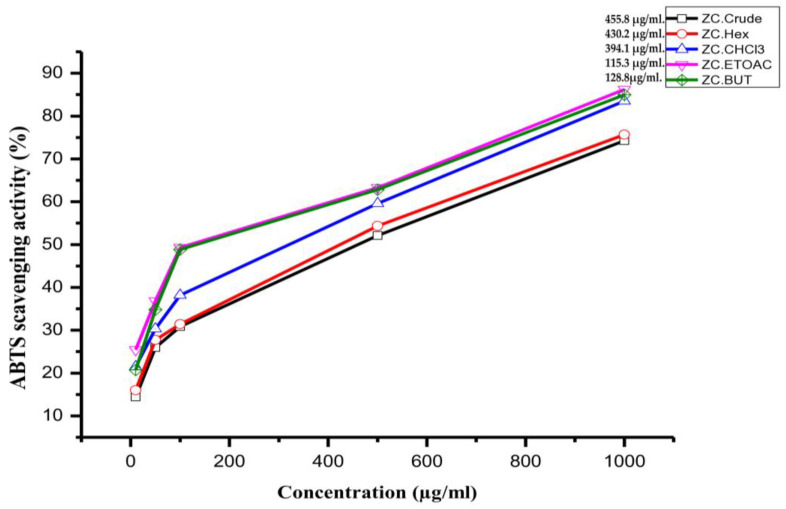
The antioxidant activity of *Z. coccineum* crude extract and fractions was measured using the ABTS scavenging activity method. Butanol is denoted as BUT, ethyl acetate is represented as EtOAC, chloroform is indicated by CHCL3, and n-hexane is referred to as Hex. Crude methanol extract is abbreviated as Crude.

The antioxidant potential of *Zygophyllum coccineum* extract and its fractions was assessed using ABTS radical scavenging assays, as illustrated in Figure 3. The results demonstrated a notable capacity of the extract to neutralize free radicals across varying concentrations. Among the tested fractions, the ethyl acetate fraction exhibited the highest antioxidant activity, with an IC50 value of 115.3 µg/mL. In comparison, the crude methanol extract displayed the lowest antioxidant activity, with an IC50 value of 455.8 µg/mL. For reference, the positive control, quercetin, demonstrated superior effectiveness, with an IC50 value of 1.7 µg/mL.

### 3.4. Cytotoxic Activity

**Figure 4:**
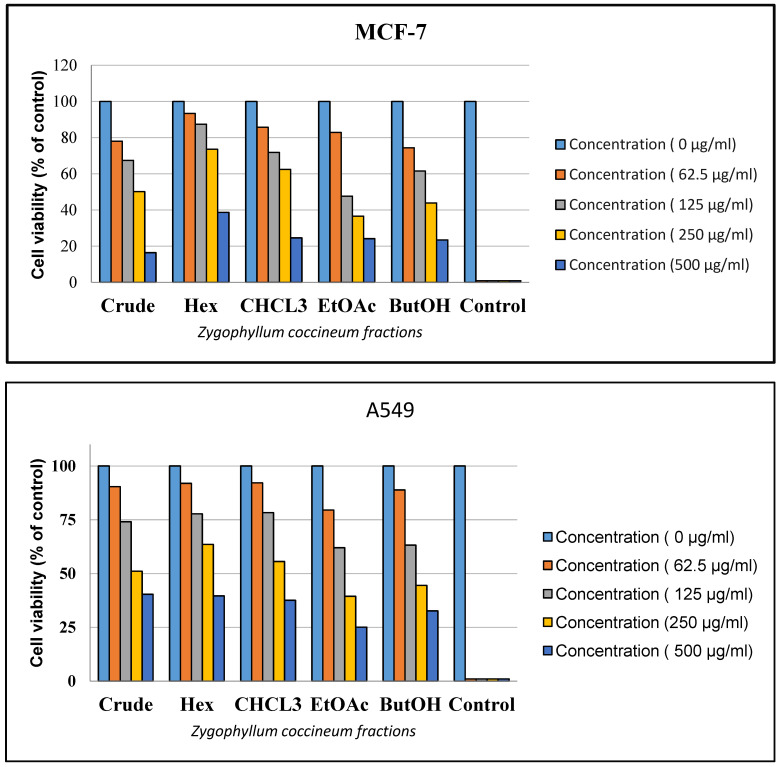
*Zygophyllum coccineum* crude extract and its fractions had an antiproliferative effect on MCF-7 and A549 cell lines. Cell viability inhibition was expressed as a percentage (of negative control) after cells were treated with varying concentrations (0, 62.5, 125, 250, and 500 µg/mL). Doxorubicin served as the positive control in this study. The data are the average ± standard deviation of three separate studies.

The cytotoxic potential of *Zygophyllum coccineum* extract and its fractions was assessed using an in vitro MTT assay. The findings, summarized in Table 3, demonstrate a dose-dependent antiproliferative effect of the fractions on various cancer cell lines, as depicted in Figure 4. Among the fractions, the ethyl acetate fraction exhibited the most pronounced cytotoxic activity, particularly against breast (MCF-7) and lung (A549) cancer cells. This fraction achieved IC_50_ values of 119.3 µg/mL for MCF-7 cells and 190.7 µg/mL for A549 cells, indicating its significant cytotoxicity. In comparison, the other fractions displayed minimal inhibitory effects on the tested cancer cell lines. Doxorubicin was used as the positive control in this study.

### 3.5. Antimicrobial Activity

The extracts derived from the studied subshrub exhibited varying degrees of inhibitory effects against the tested bacterial and fungal strains, as detailed in Table 4 and Table 5. Antimicrobial activity was evaluated by measuring the diameters of the growth inhibition zones (clear zones). The results indicated that all tested bacteria and fungi were susceptible to the extracts, with statistically significant differences (*p* < 0.05) observed in the average inhibition zone diameters among the various extracts.

The ethyl acetate extract of *Zygophyllum coccineum* demonstrated the strongest antimicrobial activity against bacterial and fungal strains, followed by the acetone extract and the hot aqueous extract. In contrast, the n-hexane, n-butane, and ether extracts exhibited moderate levels of antimicrobial activity. Notably, the cold aqueous extract showed no antimicrobial effects against any of the tested strains.

### 3.6. Antimicrobial Assay: MIC and MBC

The results summarized in Table 6 highlight the notable antimicrobial properties of the *Zygophyllum coccineum* subshrub, with its highest antimicrobial activity evident in ethyl acetate extract against both animal-derived pathogenic strains and standard reference strains. This extract consistently demonstrated potent antibacterial and antifungal properties, emphasizing its potential as a significant antimicrobial agent.

## 4. Discussion

This study highlights the therapeutic potential of *Zygophyllum coccineum*, particularly its ethyl acetate extract, which demonstrated superior antioxidant, cytotoxic, and antimicrobial properties. The phytochemical analysis revealed a rich composition of bioactive compounds, including phenolics, flavonoids, and fatty acids, which contribute significantly to the observed pharmacological effects. Phenolic compounds, such as Gallic acid derivatives, are potent antioxidants known for their ability to neutralize reactive oxygen species (ROS), thus reducing oxidative stress and preventing lipid peroxidation [17]. Flavonoids, another key group of phytochemicals identified, are effective scavengers of free radicals and metal ion chelators, supporting cellular protection and contributing to antioxidant activity [18]. The presence of fatty acids, such as n-hexadecanoic acid, further enhances the bioactivity of the extracts, particularly in antimicrobial and anti-inflammatory applications [19].

The ethyl acetate fraction exhibited the highest levels of phenolic and flavonoid content, correlating with its strong antioxidant activity, as evidenced by the low IC_50_ value in the ABTS assay. This robust activity aligns with previous studies on *Zygophyllum album* and *Zygophyllum simplex*, which have similarly demonstrated significant antioxidant potential linked to their phenolic profiles [20,21]. Polar solvents like methanol and butanol were more effective in extracting bioactive compounds, while non-polar solvents like hexane and chloroform exhibited minimal activity. Ethyl acetate, which is moderately polar, demonstrated significant efficacy in extracting compounds with antimicrobial and anticancer potential, underscoring the importance of solvent polarity in phytochemical extraction.

The cytotoxic activity of the ethyl acetate extract against A549 (lung carcinoma) and MCF-7 (breast cancer) cell lines further highlights the pharmacological significance of *Z. coccineum*. The observed cytotoxic effects, with low IC_50_ values, suggest the presence of bioactive compounds capable of inducing apoptosis and inhibiting cancer cell proliferation. These effects are likely mediated by flavonoids and phenolic acids, which are known to disrupt critical pathways in cancer cell metabolism, including mitochondrial dysfunction, DNA damage, and ROS modulation [22,23]. Similar findings in related species, such as *Z. album* and *Z. simplex*, reinforce the roles of phenolic and flavonoid compounds in anticancer activity [24,25]. Notably, non-polar fractions showed limited activity, further emphasizing the contributions of polar bioactive compounds.

The antimicrobial activity of *Z. coccineum* extracts, particularly the ethyl acetate and acetone fractions, demonstrated broad-spectrum efficacy against both Gram-positive (*Staphylococcus aureus*) and Gram-negative (*Escherichia coli*, *Salmonella typhimurium*) bacteria, as well as the fungal pathogen *Candida albicans*. This activity can be attributed to phenolic and flavonoid compounds, which are known to disrupt microbial membranes and inhibit enzymatic pathways critical for pathogen survival [26]. The identification of bioactive compounds, such as n-hexadecanoic acid and sesquiterpenes, in the GC-MS analysis supports the antimicrobial efficacy observed. These findings are consistent with prior studies on *Z. album* and *Z. simplex*, in which ethyl acetate fractions showed significant activity against various pathogenic microbes [27]. The ability of *Z. coccineum* to target both bacterial and fungal pathogens underscores its versatility and potential as a natural alternative to synthetic antibiotics, addressing the growing challenge of antimicrobial resistance (AMR).

The pharmacological properties of *Z. coccineum* extend beyond therapeutic applications. Its resilience to harsh environmental conditions and ability to thrive in arid regions position it as a sustainable source of bioactive compounds in resource-constrained settings. The use of *Z. coccineum* as a natural feed additive for livestock could reduce reliance on synthetic antibiotics, mitigating the emergence of resistant pathogens and promoting safer animal products for human consumption. Additionally, its antioxidant and cytotoxic properties open avenues for its integration into nutraceuticals and functional foods aimed at enhancing health and preventing disease.

While the findings of this study are promising, further research is essential to fully realize the therapeutic potential of *Z. coccineum*. In vivo studies are necessary to validate the observed pharmacological effects and assess the safety and efficacy of the extracts in biological systems. The isolation and structural characterization of individual bioactive compounds will enhance our understanding of their mechanisms of action and facilitate the development of targeted therapies. Moreover, exploring potential synergistic effects between *Z. coccineum* extracts and existing antimicrobial or cytotoxic agents could yield novel combination treatments with enhanced efficacy. Finally, investigating the ecological applications of *Z. coccineum*, such as its use in phytoremediation, could expand its utility beyond medicinal contexts, contributing to environmental sustainability.

## 5. Conclusions

This study highlights the significant antioxidant, cytotoxic, and antimicrobial properties of *Z. coccineum*, particularly in its ethyl acetate extract. These findings establish the plant as a promising candidate for the development of natural therapeutics targeting AMR and utilizing cytotoxic effects on the tested cancer cell lines. Future research should focus on in vivo validation, compound isolation (especially from the ethyl acetate extract), and the exploration of synergistic effects to fully realize its therapeutic potential, in addition to the need for future studies to incorporate multiple isolates from farm animals as well as corresponding ATCC strains for each bacterial species.

## Figures and Tables

**Table 1:** The total phenolic and flavonoid content found in the crude extract of *Zygophyllum coccineum*.

Shrub	Total Phenol mg GAE/g of Dry Extract	Total Flavonoid mg QE/g of Dry Extract
*Zygophyllum coccineum*	156.15 ± 0.018	18.5 ± 0.013

**Table 2:** Putative phytochemical profile of *Zygophyllum coccineum* using GC-MS.

Compound	RT (min)	Putative Compound Name	Mol Weight (amu)	Area (Ab*s)	% Abundance	Confidence Score
**1**	5.927	Undecane	156.188	249,400	0.47%	87
**2**	6.583	4H-Pyran-4-one, 2,3-dihydro-3,5-dihydroxy-6-methyl-	144.042	585,141	1.10%	72
**3**	7.835	5-Acetoxymethyl-2-furaldehyde	168.042	301,778	0.57%	35
**4**	9.642	6,8-Dioxa-3-thiabicyclo(3,2,1)octane 3,3-dioxide	164.014	3,184,450	5.99%	47
**5**	10.556	Bicyclo[4.4.0]dec-1-ene, 2-isopropyl-5-methyl-9-methylene-	204.188	302,011	0.57%	90
**6**	10.881	Bicyclo[3.1.1]heptane-2-carboxaldehyde, 6,6-dimethyl-	152.12	541,087	1.02%	38
**7**	11.225	Glutaconic acid	130.027	379,218	0.71%	43
**8**	11.613	Cyclooctene, 3-methyl-	124.125	956,690	1.80%	41
**9**	11.894	2-Propenoic acid, 2-methyl-, 1,2-ethanediylbis(oxy-2,1-ethanediyl) ester	286.142	519,131	0.98%	80
**10**	12.094	1,3-Dithiane, 2,2-dimethyl-	148.038	496,420	0.93%	41
**11**	12.338	n-Hexadecanoic acid	256.24	979,229	1.84%	99
**12**	12.551	Diazoprogesterone	338.247	535,236	1.01%	38
**13**	12.745	1,5-Cyclooctadiene, 3,4-dimethyl-	136.125	3,108,471	5.85%	58
**14**	12.883	cis-α-Bisabolene	204.188	285,721	0.54%	58
**15**	13.152	Propanoic acid, 2-methyl-, 2-[3-[(acetyloxy)methyl]oxiranyl]-5-methylphenyl ester	292.131	2,741,721	5.17%	27
**16**	13.552	Putative Cyclopentane-3′-spirotricyclo[3.1.0.0(2,4)]hexane-6′-spirocyclopentane	188.157	22,662,616	42.60%	64
**17**	13.790	Spiro[2.4]heptane, 4-methylene-	108.094	3,161,261	5.95%	58
**18**	14.202	Hexanoic acid, but-3-yn-2-yl ester	168.115	775,810	1.46%	43
**19**	14.609	Cyclohexene, 1-methyl-4-(5-methyl-1-methylene-4-hexenyl)-, (S)-	204.188	682,688	1.28%	49
**20**	14.828	Bergamotol, Z-α-trans-	220.183	1,715,991	3.23%	50

Note: The compounds listed are putative proposals based on GC-MS analysis and bioinformatic searching of libraries and databases. These compounds cannot be considered definitively identified without further purification and structural confirmation (e.g., NMR or comparison to standards).

**Table 3:** The IC_50_ values for the crude extract of *Zygophyllum coccineum*, along with its various fractions.

Cell Lines	ZC Fractions IC_50_ (µg/mL)					
	Crude	Hex	CHCl_3_	EtoAc	ButOH	Doxorubicin
A549	252.1 ± 4.2	389.6 ± 3.6	315.3 ± 4.5	190.7 ± 2.1	211.8 ± 0.8	3.52 ± 0.05
MCF-7	250 ± 2.4	418.5 ± 2.5	331.9 ± 2.5	119.3 ± 1.2	204.4 ± 3.6	2.5 ± 0.03

**Table 4:** The antimicrobial activity of solvent extracts derived from *Zygophyllum coccineum* against reference microbial strains.

Pathogens	*S. aureus* ATCC 29213	*E. faecalis* ATCC 29212	*E. coli* ATCC 25922	*S. typhimurium* ATCC 14028	*C. albicans* ATCC 60193	*C. tropicalis* ATCC 66029
Extracts						
But	0	0	11.26 ± 0.25	0	0	0
EtOAC	17.23 ± 0.25	14.3 ± 0.26	14.23 ± 0.25	12.23 ± 0.20	23.23 ± 0.25	19.23 ± 0.25
CHCL3	11.13 ± 0.15	0	0	0	0	0
Hex	10.23 ± 0.25	11.26 ± 0.25	12.26 ± 0.25	11.23 ± 0.30	0	0
CM	16.2 ± 0.26	0	15.03 ± 0.25	13.1 ± 0.2	13.2 ± 0.2	11.36 ± 0.32
E	0	10.16 ± 0.15	14.2 ± 0.2	11.16 ± 0.35	14.3 ± 0.20	13.2 ± 0.26
A	11.26 ± 0.25	0	17.33 ± 0.35	0	20.23 ± 0.32	14.46 ± 0.30
HA	11.5 ± 0.2	0	19.3 ± 0.26	12.33 ± 0.35	16.4 ± 0.2	13.5 ± 0.2
CA	0	0	0	0	0	0
C-VE (D.W.)	0	0	0	0	0	0
C+VE/Cipro	21.5 ± 0.5	18.3 ± 0.2	19.83 ± 0.76	18.37 ± 0.35	U	U
C+VE/Ny	U	U	U	U	18.17 ± 0.15	19.87 ± 0.15

Abbreviations: But = Butanol, EtOAC = Ethyl acetate, Hex = n-Hexane, CHCL3 = Chloroform, CM = Crude methanol, HA = Hot aqueous, C+VE = Positive control, Cipro = Ciprofloxacin (5 µg), Ny = Nystatin (30 µg), U = Unutilized.

**Table 5:** The antimicrobial activity of *Zygophyllum coccineum* extracts against various isolated animal microorganisms.

Extracts	EtOAC	Hex	CHCL3	CM	E	A	But	HA	CA	C-VE (D.W)	C+VE/Cipro	C+VE/Ny
Pathogens												
*Ps. aeruginosa*	22.3 ± 0.26	0	0	0	0	0	0	12.23 ± 0.25	0	0	31.23 ± 0.25	U
*E coli*	14.26 ± 0.25	11.16 ± 0.15	16.26 ± 0.25	15.46 ± 0.45	13.16 ± 0.30	11.4 ± 0.4	10.4 ± 0.26	18.4 ± 0.3	0	0	19.23 ± 0.21	U
*E. faecium*	12.26 ± 0.25	0	10.16 ± 0.30	13.16 ± 0.15	0	0	11.4 ± 0.03	0	0	0	35.3 ± 0.3	U
*S. typhimurium*	11.4 ± 0.26	10.26 ± 0.25	0	12.46 ± 0.45	10.16 ± 0.15	0	0	11.16 ± 0.30	0	0	30.13 ± 0.32	U
*K. pneumoniae*	18.26 ± 0.25	0	0	15.23 ± 0.22	14.4 ± 0.26	13.16 ± 0.20	0	14.16 ± 0.15	0	0	34.2 ± 0.2	U
*B. cereus*	15.4 ± 0.4	0	11.16 ± 0.15	17.4 ± 0.03	13.26 ± 0.25	19.4 ± 0.26	0	19.46 ± 0.45	0	0	34.1 ± 0.2	U
*Streptococcus pyogenes*	13.26 ± 0.25	0	0	0	11.46 ± 0.45	0	12.16 ± 0.30	10.26 ± 0.25	0	0	R	U
*S. aureus*	16.26 ± 0.25	11.23 ± 0.22	0	0	0	12.4 ± 0.26	0	13.3 ± 0.26	0	0	25.37 ± 0.40	U
*C albicans*	22.4 ± 0.26	0	0	13.4 ± 0.03	14.46 ± 0.45	20.16 ± 0.15	0	15.16 ± 0.20	0	0	U	16.33 ± 0.31

Abbreviations: But = Butanol, EtOAC = Ethyl acetate, Hex = n-Hexane, CHCl3 = Chloroform, CM = Crude methanol, HA = Hot aqueous, C+VE = Positive control, Cipro = Ciprofloxacin (5 µg), Ny = Nystatin (30 µg), U = Unutilized.

**Table 6:** The minimum inhibitory concentration (MIC) and minimum bactericidal/fungicidal concentration (MBC/MFC) of the ethyl acetate extract derived from *Zygophyllum coccineum*.

Examined Strains	Concentrations of *Ethyl acetate* Extract (mg/mL)							
	5	25	50	75	100	150	MIC	MBC/MFC
*Ps. aeruginosa*	_	_	_	_	_	_	5 mg/mL	25 mg/mL
*E coli*	+	+	_	_	_	_	50 mg/mL	75 mg/mL
*E. faecium*	++	+	_	_	_	_	50 mg/mL	75 mg/mL
*S. typhimurium*	++	+	_	_	_	_	50 mg/mL	75 mg/mL
*K. pneumoniae*	_	_	_	_	_	_	5 mg/mL	25 mg/mL
*B. cereus*	+	_	_	_	_	_	25 mg/mL	50 mg/mL
*Streptococcus pyogenes*	+	+	_	_	_	_	50 mg/mL	75 mg/mL
*S. aureus*	+	_	_	_	_	_	25 mg/mL	50 mg/mL
*C. albicans*	_	_	_	_	_	_	5 mg/mL	25 mg/mL
*S. aureus ATCC 29213*	_	_	_	_	_	_	5 mg/mL	25 mg/mL
*E. faecalis ATCC 29212*	+	+	_	_	_	_	50 mg/mL	75 mg/mL
*E. coli ATCC 25922*	+	+	_	_	_	_	50 mg/mL	75 mg/mL
*S. typhimurium ATCC 14028*	++	+	_	_	_	_	50 mg/mL	75 mg/mL
*C. albicans ATCC 60193*	_	_	_	_	_	_	5 mg/mL	25 mg/mL
*C. tropicalis ATCC 6602*	+	+	_	_	_	_	50 mg/mL	75 mg/mL

(+) indicates a turbid condition characterized by microbial growth, (++) signifies a very turbid state associated with high levels of microbial growth, while (_) denotes a lack of turbidity, indicating the absence of microbial growth.

## Data Availability

The original contributions presented in this study are included in the article. Further inquiries can be directed to the corresponding author.
